# Post-Crackdown Effectiveness of Field-Based Forest Law Enforcement in the Brazilian Amazon

**DOI:** 10.1371/journal.pone.0121544

**Published:** 2015-04-15

**Authors:** Jan Börner, Krisztina Kis-Katos, Jorge Hargrave, Konstantin König

**Affiliations:** 1 Center for Development Research, University of Bonn, Bonn, Germany; 2 Center for International Forestry Research, Bogor, Indonesia; 3 Institute for Economic Research, University of Freiburg, Freiburg, Germany; 4 The Boston Consulting Group, São Paulo, Brazil; 5 Integrative Research Institute on Transformations of Human-Environment Systems, Humboldt-Universität zu Berlin, Berlin, Germany; University of Brasilia, BRAZIL

## Abstract

Regulatory enforcement of forest conservation laws is often dismissed as an ineffective approach to reducing tropical forest loss. Yet, effective enforcement is often a precondition for alternative conservation measures, such as payments for environmental services, to achieve desired outcomes. Fair and efficient policies to reducing emissions from deforestation and forest degradation (REDD) will thus crucially depend on understanding the determinants and requirements of enforcement effectiveness. Among potential REDD candidate countries, Brazil is considered to possess the most advanced deforestation monitoring and enforcement infrastructure. This study explores a unique dataset of over 15 thousand point coordinates of enforcement missions in the Brazilian Amazon during 2009 and 2010, after major reductions of deforestation in the region. We study whether local deforestation patterns have been affected by field-based enforcement and to what extent these effects vary across administrative boundaries. Spatial matching and regression techniques are applied at different spatial resolutions. We find that field-based enforcement operations have not been universally effective in deterring deforestation during our observation period. Inspections have been most effective in reducing large-scale deforestation in the states of Mato Grosso and Pará, where average conservation effects were 4.0 and 9.9 hectares per inspection, respectively. Despite regional and actor-specific heterogeneity in inspection effectiveness, field-based law enforcement is highly cost-effective on average and might be enhanced by closer collaboration between national and state-level authorities.

## Introduction

Annual deforestation in the Brazilian Amazon is down from 27772 km^2^ in 2004 to 4656 km^2^ in 2012—the lowest annual forest loss since annual satellite-based deforestation data became available in 1989. Evaluations of Brazil’s anti-deforestation strategy, the PPCDAM, suggests that a combination of mostly regulatory policy instruments, such as protected area declaration, law enforcement operations, and embargos on blacklisted districts and farms in the Amazon have been key success factors [1–4]. Arima et al. [5], for example, find that forest conservation policies have avoided 10,653 km^2^ of deforestation in a subgroup of priority districts over the period from 2009 to 2011.

Understanding how the specific Brazilian policy mix has caused land users in the Amazon to deforest less, can help countries concerned with Reducing Emissions from Deforestation and Degradation (REDD+), a planned international climate policy mechanism, to conserve forests more effectively. It can also contribute to fine-tune the Brazilian anti-deforestation strategy in the light of increased legal scope for deforestation after a recent forest law reform [6, 7].

Counterfactual-based evaluations of specific elements of Brazil’s anti-deforestation policy mix, such as the expansion of the protected areas network, the role of environmental fines, and the effect of conditioning credit access on compliance with forest law are slowly emerging in the peer-reviewed literature. Soares-Filho et al. [8] attribute 44% of the drop in deforestation rates between 2004 and 2006 to a general agricultural slowdown and 37% specifically to the expansion of the regions protected area network. The remaining 18%, the authors argue, could be explained by other policy changes. In a study at the municipal scale covering the years 2002 to 2009, Hargrave and Kis-Katos [9] find that a 1% increase in the fining intensity (measured by total fines per deforested area) in a municipality has reduced deforestation by on average 0.2%.

Research on actor-specific temporal deforestation patterns in the Brazilian Amazon shows, moreover, that deforestation dynamics are shifting from large to smaller-scale clearings [10] and that reductions in annual forest loss predominantly accrued on large land holdings [11].

**Fig 1:**
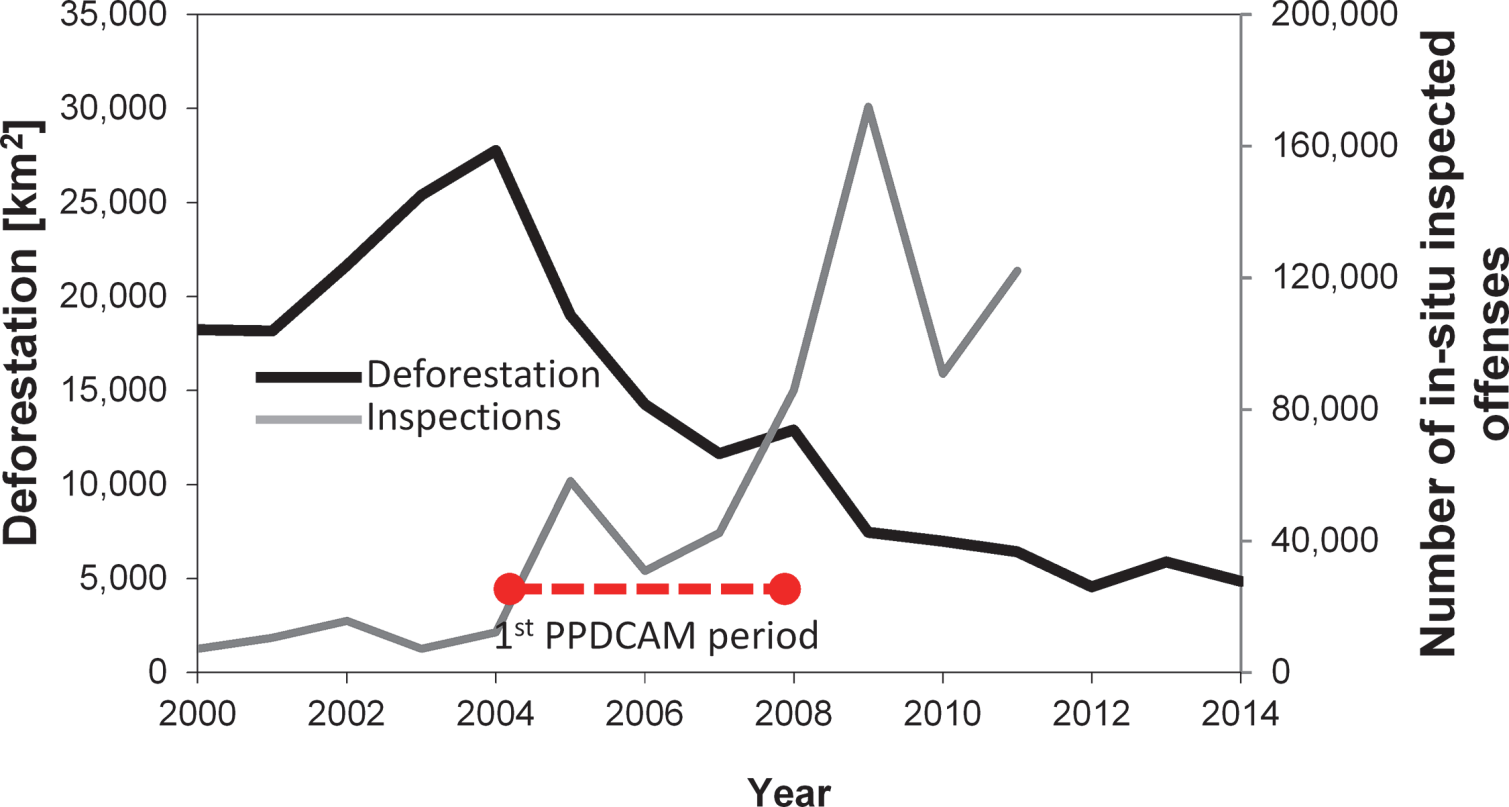
Annual deforestation and number of inspected offenses from 2000–2011.

Recent research thus points to policy change as one of the key factors that have brought deforestation rates down to current levels. Knowledge gaps, however, remain with respect to the underlying mechanisms that have ultimately affected land use decisions in the region and how these effects may vary at sub-regional scales. Here we address this knowledge gap by studying a key incentive delivery mechanism of the Brazilian anti-deforestation strategy: field-based enforcement missions. Our interest lies in understanding by how much and where field-based enforcement missions are deterring deforestation at a point in time when annual forest loss had stabilized close to the currently low levels in what could be called the “post- 1^st^ PPCDAM period” (see Fig. 1). The second PPCDAM has been implemented between 2009 and 2011 and the third PPCDAM runs until 2015.

Field-based enforcement, a major PPCDAM component, links remotely-sensed deforestation patches to potentially liable individuals or groups of land users. Once liability is established, offenders may face significant disincentives in the form of multiple layers of legal coercion, such as confiscation of assets and monetary fines or cross-compliance mechanisms like conditional access to credit and commercialization channels. We estimate the average deterrence effect of field based enforcement missions (or inspections) based on 15432 geo-referenced field contacts of agents from Brazil’s environmental protection agency (IBAMA) during the period 2009–2010 on total deforestation and deforestation patterns in subsequent years. Using a matching approach, we identify spatial grid cells on a scale of 10x10km^2^ that have comparable observable characteristics to those visited by IBAMA and use them as controls. We find that field-based enforcement operations have not been universally effective in deterring deforestation during our observation period. Differences in effectiveness exist across states and scale of deforestation. On average inspections have been ineffective in deterring small-scale deforestation and have significantly reduced absolute deforestation in only two out of six states.

The remainder of the paper is structured as follows. In section 2, we develop a simple conceptual framework motivating our hypotheses on the effects of field-based enforcement operations in the context of the Brazilian Amazon. Section 3 describes our study area and documents the data used in this study. Section 4 describes the empirical strategy and section 5 presents our results. In section 6 we discuss our findings and implications for forest conservation policy.

## Conceptual Framework and Hypotheses

Becker’s [12] major contribution to the theory of law enforcement has strongly influenced economic thinking about optimal law enforcement, including law enforcement in the conservation sector [13–15]. In the context of illegal deforestation, Becker’s basic model can be restated as follows:

def=f(π−pF) (eq. 1)

Here *def*, the total area that a representative land user deforests per year, is a function of the expected per-hectare net economic return to deforestation *π* minus the expected value of the punishment for illegal deforestation. The expected legal costs of deforestation depend on the probability of deforestation being detected and effectively sanctioned *p* and the official per-hectare fine for illegal deforestation *F*. The combined effect of the legal disincentive (e.g. a fine) and the effectiveness of its delivery mechanism (enforcement probability) is the actual deterrence effect. If deterrence is higher than the expected return to deforestation, land users will comply with the law. However, deforestation will occur wherever expected returns exceed deterrence. If enforcement is costly, as is the case with field-based enforcement, Becker concluded that fines should be set relatively high to achieve compliance. Several later extensions of the model, however, identified conditions, under which high fines are non-optimal substitutes for low levels of enforcement probability [15, 16]. Polinsky and Shavell [17], for example, suggest that fairness considerations can lead social planners to reduce fine levels and increase enforcement probability.

In Brazil, monetary fines tend to be of low relevance historically. Between 2008 and 2010, only 0.3% of the total value of fines issued by IBAMA had been actually collected by the authorities [18]. The increased deterrence effect of anti-deforestation policies in the Amazon region thus has to be the result of two complementary measures. First, a surge in field presence (see Fig. 1) resulting in higher enforcement probabilities (*p*); and second, an increase in the total cost of being held liable for illegal deforestation in the form of alternative coercion mechanisms equivalent to *F* in Equation 1. Clarke et al. [19], for example, note that confiscation of illegally acquired goods can serve as a fine-equivalent coercive measure. Administrative cross-compliance measures that condition access to public support measures on environmental compliance can also complement monetary sanctions in complex law enforcement systems [20]. With this in mind, Equation 1 modifies such that:

def=f(π−p(qF+C)) (eq. 2)

where *q* is the actual share of fines collected and *C* is the monetary equivalent of alternative coercive measures, such as asset confiscation or bans on credit access for farmers who have illegally cut forests. Where *q* is low, as in Brazil, *C* must increase such that *p*(*qF* + *C*) ≥ *π* for enforcement to become an effective deterrent.

In a review of the enforcement literature, Robinson et al. [13] emphasized the importance of spatial dynamics in the enforcement of environmental policies. For example, even if fines are high formally, a policy’s deterrence effect may be low in remote locations, where budget constraints limit the enforcement agency’s ability to confront potential offenders with regular field presence, lowering *p* [21, 22].

Robinson et al., however, also note that high fines may induce costly avoidance behavior among offenders. In fact, based on a spatial analysis of deforestation patterns in the Brazilian Amazon, Rosa et al. [10] suggest that deforestation patch size is shifting from large to small and has become more difficult to detect over time, allegedly in response to law enforcement operations that have targeted large-scale deforestation in the past. To see how this could happen consider the following: without an environmental policy incentive, *p F* in Equation (1) is zero and each land user’s total deforestation would depend on *π* alone. If, however, an environmental policy was in place and both *π* and *p* were increasing in deforestation patch size *s*, this would put an upper limit on the optimal deforestation patch size. It would be not economically viable to further increase the size of a deforestation patch as soon as following condition holds:

$$\frac{\partial \pi }{\partial s}<\frac{\partial p}{\partial s}\left(qF+C\right)$$ (eq. 3)

that is, once the marginal economic gain from additional deforestation in a given geographic area is smaller than the expected increase in the value of fines and alternative coercive measures due to an increased probability of detection.

Our empirical analysis first tests whether documented field inspections in a given location and year do in fact result in lower deforestation in that location the following year. In a second step, we test whether the inspection also affects the distribution of deforestation patch sizes in the location in subsequent years.

## Data and Study Area

The data sources used in this study are summarized in Table 1.

**Table 1:** Data sources.

Data/Variable	Resolution	Source(s)
Location and type of IBAMA’s field inspections (2009–2010)	Spatial point data	IBAMA
Deforestation and cloud cover (1997–2011)	Polygon shape files	INPE-PRODES and DETER accessed 29.4.2013: http://www.obt.inpe.br/prodes/index.php
Indigenous territories	Polygon shape files	MMA-Mapas
Protected areas	Polygon shape files	MMA-Mapas
Average annual precipitation	Polygon shape files	MMA-Mapas
Land cover types (2007)	Polygon shape files	INPE-TerraClass
Socio-economic data	Municipal level	IBGE Agricultural Census 2006
Distance to municipal centers	20x20 km grid cells	Börner et al. [23]

**Fig 2:**
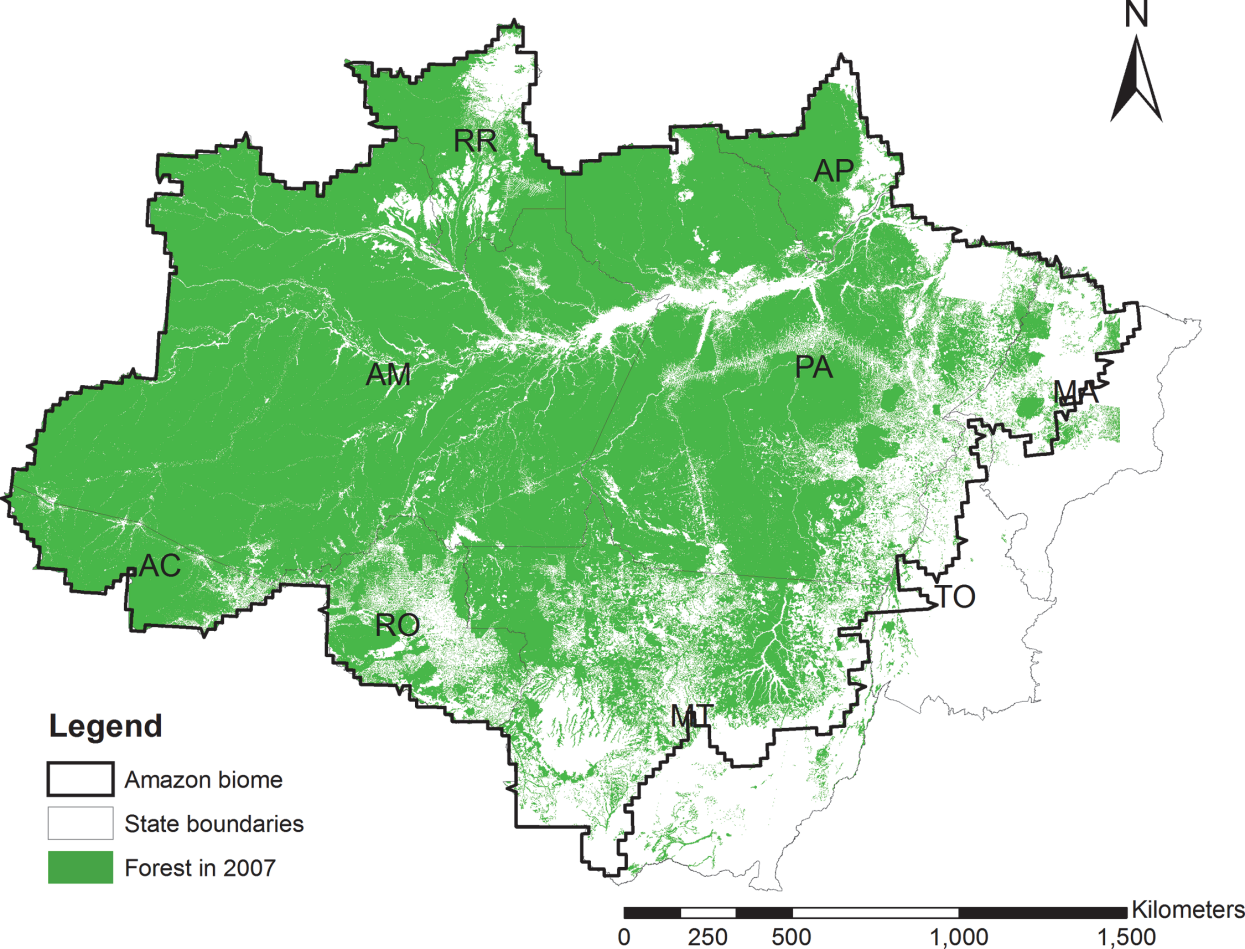
Study area and 2007 forest cover in the Legal Amazon.

Our study area represents the Amazon Biome and excludes a considerable share of the southeastern Legal Amazon region (Fig. 2), most of which has no or only negligible forest cover according to the National Space Research Institute (INPE).

We organized the spatial point data on documented field inspections issued by IBAMA in the period from 2009–2010 according to the observation period of INPE’s monitoring system PRODES, resulting in two consecutive observation periods. Inspections realized between August 1, 2008 and July 31, 2009 represent *observation period 2009*, whereas inspections between August 1, 2009 and July 31, 2010 represent the *observation period 2010*. We distinguish between offenses that fall into the categories “flora”, “fauna”, and “other”, with illegal deforestation being included in the “flora” category (Table 2). “Flora” also includes illegal logging. “Fauna” mainly includes illegal hunting and fishing, whereas “other” includes transportation of illegal forest products.

**Table 2:** Counts of geo-referenced inspections across categories and observation periods.

Category	Observation period 2009	Observation period 2010
Precise flora	2893	2026
Precise fauna	147	133
Precise other	856	702
Imprecise flora	2797	2708
Imprecise fauna	366	295
Imprecise other	1219	1290
***Total***	***8278***	***7154***

Spatial point data on the location of environmental offenses provided by IBAMA consists of precisely and imprecisely measured inspection points. Precise points refer to the exact geographic location of the inspection. Imprecisely measured points lack exact GPS coordinates and can only be assigned to the municipalities in which the inspection took place. Table 2 provides an overview of the distribution of inspection types across categories and observation periods.

Annual deforestation data for the Amazon is subject to considerable measurement errors caused by cloud cover. If cloud cover prevents the detection of a deforestation patch in one year, the Landsat-based PRODES monitoring system may over-report deforestation in the subsequent year. The more often a satellite passes over a region, the higher the probability of detecting deforestation in the presence of clouds. To alleviate measurement errors, we thus merged PRODES data for the years 2009–2011 with the more frequently available, but less precise, Modis-based DETER data. If DETER detected an additional deforestation polygon in one year, we eliminated the respective polygon from the PRODES image in the subsequent year.

To measure the deterrence effect of field-based inspections, we organized our data in a spatial grid with 20x20 km cell size covering the study area (Fig. 2). A slightly lower resolution 25x25 km grid has previously been used by Aguiar et al. [24] to study land use determinants in the Brazilian Amazon. In evaluations of protected area effectiveness, many studies have used spatially more detailed data sets with resolutions of less than one hectare [8, 25].

Our intermediate choice of grid resolution is justified by two reasons. The first reason is related to the nature of the intervention we intend to evaluate. Field inspections are measured as GPS point coordinates and, hence, the spatial boundaries within which the deterrence effect is expected to materialize are undefined. This is different from evaluating a protected area, where spatial pixels can be assigned to treatment and control groups based on protection status. We thus have to allow for a sufficiently large space around inspection points that we define as potential treatment area. The second reason for adopting a relatively coarse spatial resolution relates to econometric issues. Some of the socioeconomic variables used in our econometric analysis are measured at aggregate municipality scale. At high spatial resolution, these variables would introduce high within-cluster error correlation and thus biased standard errors [26]. While we rely on commonly accepted procedures to control for municipal-level error correlation, adopting a higher spatial resolution tends to exacerbate the problem apart from potentially introducing higher levels of spatial correlation [27].

Clearly, in our case the choice of grid resolution involves some degree of subjectivity. To address this, we allow for treatment and dependent variables to be measured at varying resolutions inside each 20x20 km grid cell and assess the sensitivity of the results to the scale of measurement.

We merged the municipal level variables to the same data set with grid cells being assigned to municipalities according to a maximum area criterion. The spatial location of deforestation patch centroids is used as a criterion to assign deforestation patches to grid cells. The resulting dataset has 11,181 observations (grid cells) in each observation period (see Table 3). Observation periods are pooled for all analyses.

**Table 3:** Variables used in analyses.

	Unit	Resolution	Mean	Std.dev
**Treatments**				
Inspections 2009 (precise)	Units	20x20 km	0.26	1.88
Inspections 2010 (precise)	Units	20x20 km	0.37	3.20
**Dependent variables**				
Deforestation 2011	hectares	20x20 km	50.24	160.05
Deforestation 2010	hectares	20x20 km	66.51	192.59
Deforestation 2009	hectares	20x20 km	56.40	162.18
Large-scale deforestation 2011	% of total	20x20 km	9.87	23.89
Large-scale deforestation 2010	% of total	20x20 km	12.59	2
Large-scale deforestation 2009	% of total	20x20 km	12.91	27.58
**Covariates**				
Inspections 2009 (imprecise)	Units	20x20 km	0.41	5.19
Inspections 2010 (imprecise)	Units	20x20 km	0.40	4.84
Sum of fines (2002–2008)	Units	Municipality	23.28	34.30
Accumulated deforestation 2008	hectares	20x20 km	5691.96	9948.13
Forest 2007	% cover	20x20 km	74.63	31.49
Clouds 2011	% cover	20x20 km	7.03	17.08
Clouds 2010	% cover	20x20 km	9.34	18.81
Clouds 2009	% cover	20x20 km	5.25	13.41
Distance to district center	hours	20x20 km	19.16	19.31
Blacklisted district	0 or 1	Municipality	0.20	0.40
Indigenous land	% cover	20x20 km	24.15	39.18
Protected area	% cover	20x20 km	25.12	39.32
Settlement	% cover	20x20 km	5.69	17.51
Mean annual precipitation	mm	20x20 km	2222.54	238.01
Pasture	% of total	Municipality	8.02	17.05
Agriculture	% of total	Municipality	1.02	4.76
Share of formal land owners	%	Municipality	68.34	24.59
Share of smallholders	%	Municipality	21.32	18.65
Number of tractors	Units	Municipality	95.08	166.62

## Methods and Empirical Strategy

Any empirical evaluation of the Brazilian field-based forest law enforcement strategy has to deal with the fact that enforcement operations are not randomly allocated in space. Inspections tend to concentrate where deforestation has historically been high, i.e. inspections may reduce deforestation, but deforestation also results in higher inspection rates (endogeneity). A naïve comparison of locations with and without inspections will thus tend to suggest a false positive effect of enforcement on deforestation. Similar problems of selection bias are well described in the literature evaluating the effectiveness of protected areas, which tend to be intentionally located in remote places with below average deforestation pressure [25, 28].

In their municipal level panel data analysis, Hargrave and Kis-Katos (2013) address the endogeneity of inspection intensity by two complementary instrumental variables strategies: using state level inspection intensity to instrument for municipal inspections, and instrumenting changes in environmental fining intensity with past levels of deforestation and environmental fines in a dynamic panel framework. Assunção et al. [29] exploit the fact that the spatial allocation of field inspections is correlated with cloud cover and estimate the effect of inspections in an instrumental variable approach. Both studies find significant negative effects of inspections on deforestation at the municipal scale.

With an average size of roughly 6700 km^2^, municipalities in the Brazilian Amazon are comparatively large administrative units. At the sub-municipal scale, the deterrence mechanism of field-based enforcement has not been studied yet. If the mere presence of enforcement activities in a municipality is enough to deter deforestation independent of their spatial distribution, we would expect little additional insight from a spatially explicit analysis. If, however, land users responded primarily to inspections in their immediate proximity, significant implications arise for the operational costs and design of enforcement strategies [23].

In the absence of consistent information on individual property boundaries, we have chosen a grid based representation of the Brazilian Amazon region for our analysis (see previous section). If field inspections have a spatially explicit incremental deterrence effect on illegal deforestation, an additional inspection in a given grid cell and year should on average result in less deforestation in that cell in the subsequent year. We estimate the average treatment effects relying on a matching approach. Matching has been successfully applied to evaluate a wide range of conservation initiatives [25, 28, 30]. The reliability of matching as an evaluation method hinges on the assumption of unconfoundedness [31]. In the context of our analysis, this requires the spatial allocation of inspections to be unaffected by unobserved selection criteria.

Once in the field, IBAMA officials target deforestation and other forest law infractions primarily based on remote sensing information. However, the spatial allocation of enforcement teams is the result of a prior operational planning process that prioritizes zones with a high historical deforestation record [23]. In addition, the number of enforcement teams and field operation locations is limited by budget constraints. We simulate this allocation mechanism by including historical deforestation and travel distance along with other covariates that are known to affect deforestation and forest degradation patterns in our matching analysis.

We use a matching algorithm that implements nearest-neighbor matching with replacement, i.e. control observations can be used more than once to form pairs with treated observations. We use the ‘Matching’ package for the statistical software R [32, 33]. Multiple matched control observations and the matched data are appropriately weighted. Nearest-neighbors are typically identified based on distance measures, but there are no *a priori* rules for the selection of a distance measure [34]. Here we compare two widely used measures, the Mahalanobis distance and the propensity score, and choose the one that results in the best balance among covariates after matching.

In a spatially explicit matching analysis of protected areas in Mexico, Honey-Roses et al. [35] demonstrate that the failure to control for spatial effects in the evaluation of conservation policies can result in considerable evaluation bias. Of specific interest for our analysis are spatial dependence and neighbor (or spatial treatment spillover) effects [25, 36]. Spatial dependence results from the fact that the characteristics of one grid cell tend to be correlated with the characteristics of neighboring grid cells. Spatial treatment spillover effects occur if inspections in one grid cell exert a deterrence effect on land users in neighboring grid cells. Spatial spillover effects, if unaccounted for, lead to an underestimation of the deterrence effect of any localized environmental policy since neighboring control regions would possibly affected by deterrence as well.

Precisely (geographically explicitly) measured inspections in t (see Table 2) represent our key treatment variable. Our main dependent variable is defined as the yearly change in deforestation, calculated as deforestation in period t+1 minus deforestation in period t. We also investigate deforestation in t+1 and the share of large-scale deforestation patches in total deforestation in t+1 as alternative dependent variables.

To address spatial dependence, we introduce a spatially and temporally lagged variable that characterizes a given grid cell in terms of average deforestation in all eight neighboring cells as a matching covariate [35]. To address spatial treatment spillover effects, we create two matched data sets (with and without direct neighbors). Excluding direct neighbors potentially reduces the quality of matches but at the same time mitigates problems of treatment spillovers to control grid cells. To gauge the size of such spillovers, we report results for analyses with and without excluding neighbors.

We include further controls that (a) affect the measurement of our dependent and treatment variables, (b) are known to be associated with land use decisions, and (c) are considered by IBAMA in the field operation planning process. Table 3 documents all variables used in our analysis.

Cloud cover in all observation periods is included to correct for potentially significant deforestation measurement errors (see previous section). To deal with imprecisely measured treatments, we divide the number of imprecisely measured inspections that occurred in a municipality by the size of that municipality. The resulting variable represents the average probability of receiving an inspection per square kilometer and is the same for each grid cell in a given municipality.

The presence of indigenous lands or protected areas can be expected to reduce direct deforestation pressure [8, 28, 37], while settlements or various measures of agricultural activity (the size of pastures, cropland) might put additional pressure on the remaining forest [38]. Enforcement practices also sometimes differ among these and other public and private land categories.

Deforestation has often been found to correlate with distance to urban centers, precipitation patters, and demographic characteristics [9, 24, 39, 40]. Distance to urban centers also affects the operational costs of field-based enforcement and thus comes to be an important determinant of spatial enforcement patterns [23].

Pacheco [41] and Godar et al. [42] identified actor types and related locally prevalent production systems as important land use determinants, which is why we also control for major land cover types (crops versus pastures), and the shares of smallholders as well as officially titled landholdings.

To control for districts which experienced specific coercive environmental policy action, we introduce a dummy variable for municipalities that were embargoed by the federal government after 2007 in response to extremely high deforestation rates. Embargoed municipalities are the ones with the highest forest loss in the Amazon and the embargo limits commercialization options and access to rural credit, among other cross-compliance measures [1]. For the same reason we also include the accumulated number of inspections that were recorded in each municipality during the period 2002–2008 as a district level covariate. Additional matching covariates that are known to influence IBAMA’s operational planning process include deforestation in the treatment year and before as well as the type of deforestation, here represented by the share of large scale deforestation in the treatment year.

## Results

**Fig 3:**
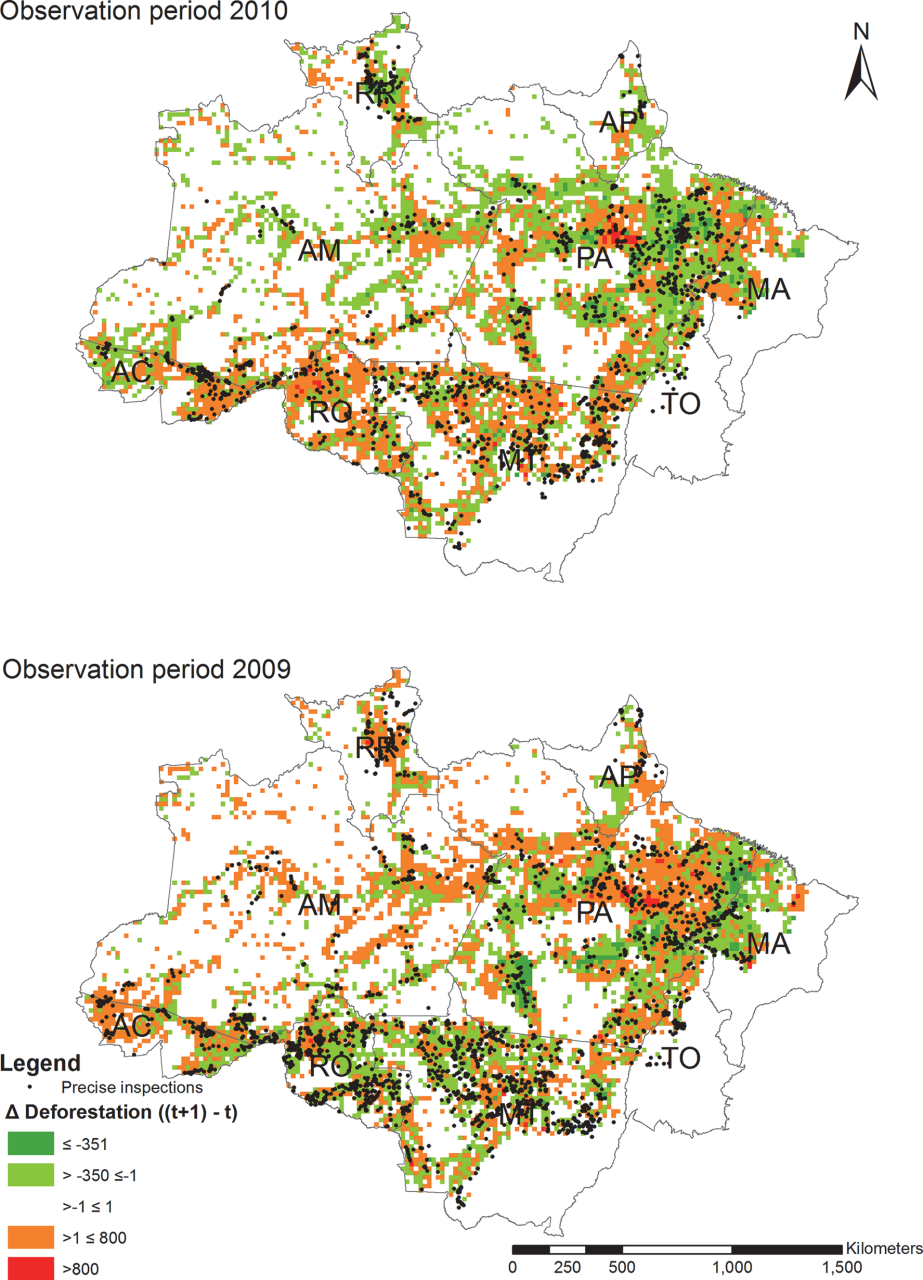
Inspection locations and changes in deforestation for observation periods 2009 (lower panel) and 2010 (upper panel).

Fig. 3 illustrates the spatial distribution of precisely measured inspections for the observation periods 2009 and 2010. Black dots represent precisely measured inspections. In red colored grid cells, deforestation was higher in the subsequent year, whereas green cells suggest reductions in forest loss. For the 2010 observation period, we observe a cluster of grid cells with reductions in deforestations in the state of Pará (PA) that coincides with a concentration of inspections. In the states of Mato Grosso (MT) and Rondônia (RO) on the other hand, inspections are rather scattered and deforestation seems to have increased in most grid cells in the subsequent year. Somewhat opposite patterns arise in the 2009 observation period. Based on a pure visual inspection, however, patters in most other states are less clear.

### Balance before and after matching

**Fig 4:**
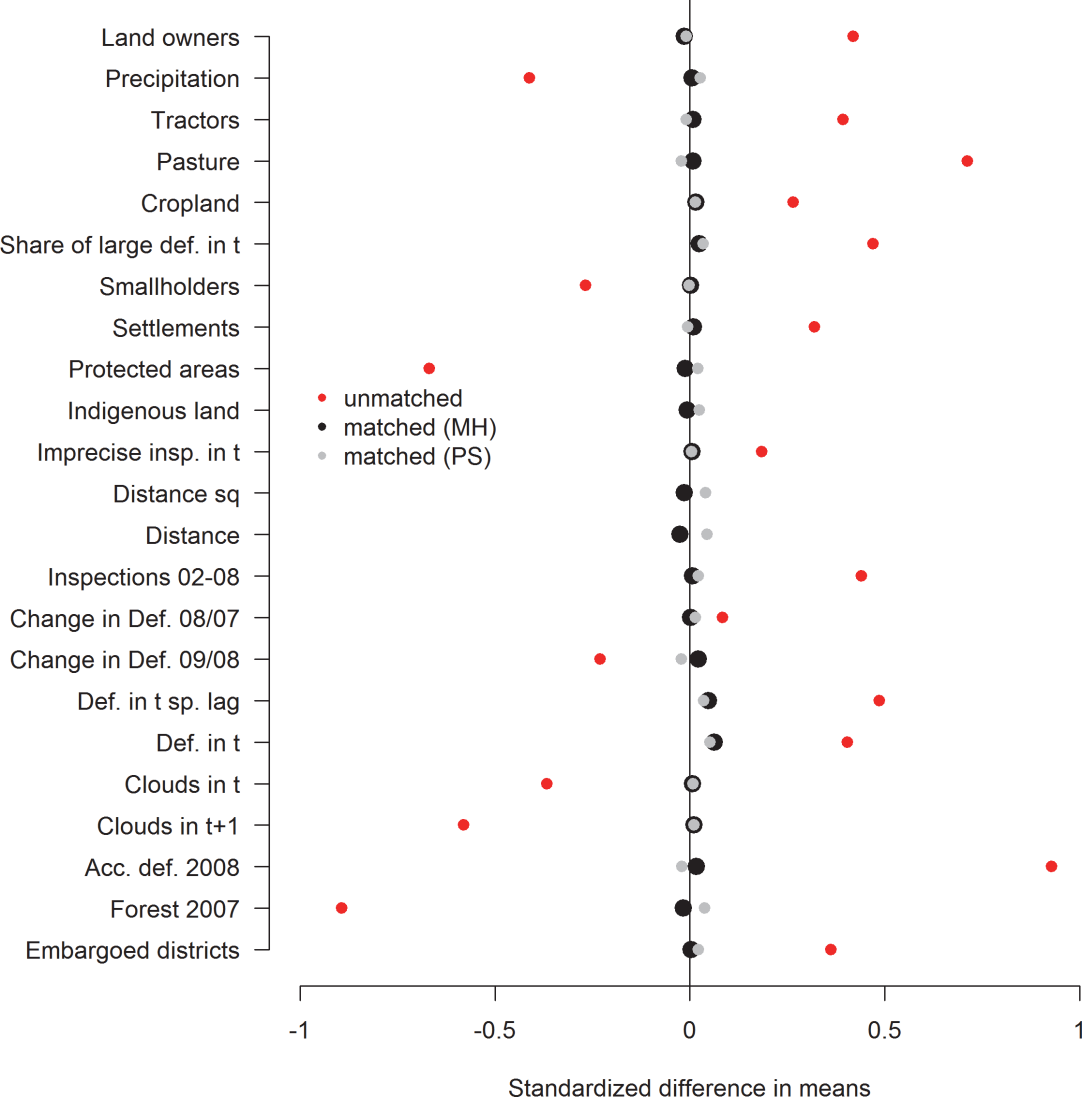
Balance before and after matching without direct neighbors using alternative distance measures.

Fig. 4 depicts standardized differences in means before and after matching without direct neighbors and compares propensity score with Mahalanobis distance matching. Visual inspection suggests a better overall balance for Mahalanobis distance matching. Before matching, comparison of means T-tests indicate significant differences between treated and control groups for all covariates. After matching differences of means remain significant at the 10% level for one covariate (deforestation in t) independent of the matching method. Given the superior overall balance, we use Mahalanobis distance matching for all subsequent analyses.

### Average effects of inspections

**Fig 5:**
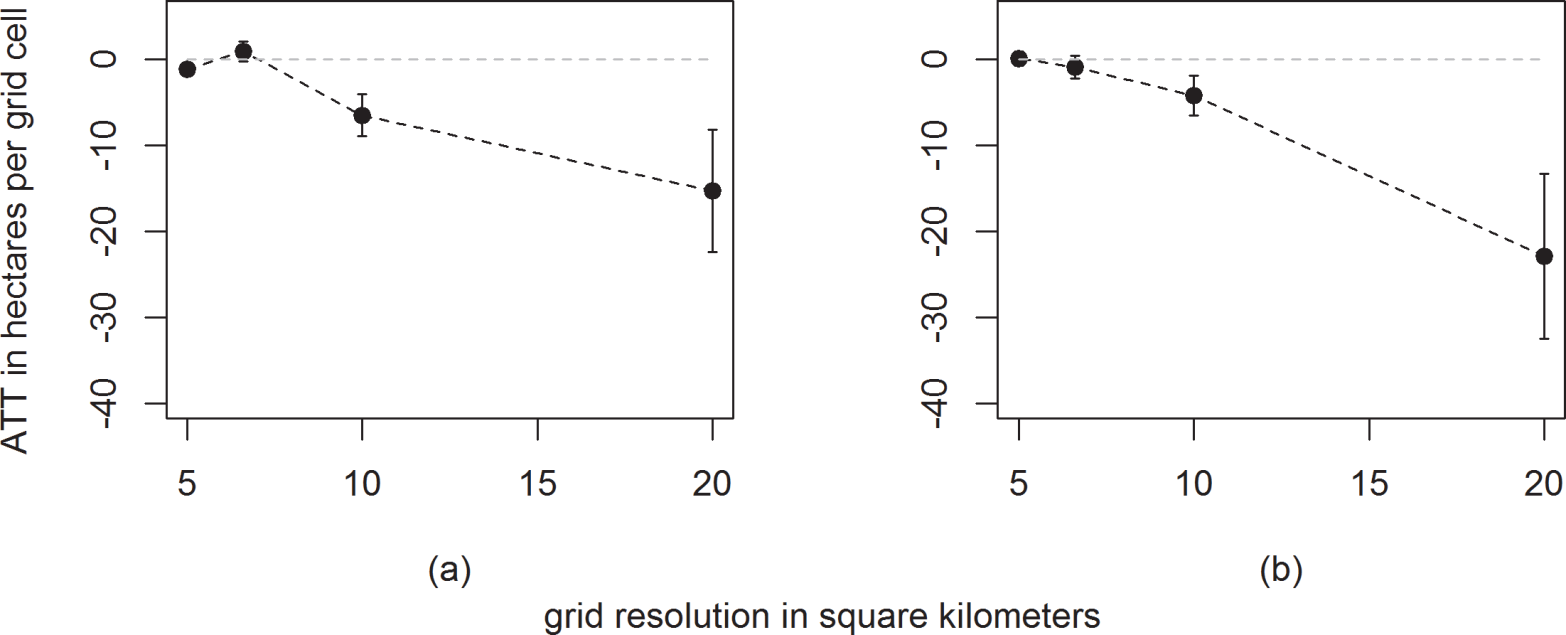
Average treatment effect on the treated (ATT) at different grid resolutions with (a) and without (b) direct neighbors. Error bars depict Abadie-Imbens (AI) standard errors.

Fig. 5 depicts average treatment effect on the treated (ATT) estimates after Mahalanobis distance matching for various grid resolutions, with and without direct neighbors among the group of potential control grid cells. The dots show point estimates for the ATT for grid resolutions of 5x5, 6.6x6.6, 10x10 and 20x20 km^2^, the corresponding error bars depict Abadie-Imbens standard errors [43]. Total absolute deforestation measured per grid cell decreases proportionally as grid cell resolution increases and ATT estimates of inspection effectiveness follow this trend. At grid resolutions beyond 10x10 km^2^, the effect of inspections on deforestation becomes small and eventually insignificant, also because spatial spillover effects become more likely as resolution increases. Moreover, at high resolutions, precisely measured inspection points are more likely to fall into a grid cell next to the cell where the actual offense was measured, thus artificially separating the inspection from the actual offense.

Comparing ATT estimates with and without direct neighbors, we find the latter are somewhat smaller, but not significantly so. We would be worried about spatial spillover effects only if ATT estimates increased significantly after omitting direct neighbors. For all subsequent analyses we adopt a 10x10 km^2^ grid resolution, because it allows us to draw on a larger number of control cells that are similar to treated cells in terms of temporally and spatially lagged deforestation estimates. At the same time, this scale is large enough to be less affected by measurement issues than the finer scales

Table 4 reports ATT estimates for alternative dependent variables and subsets of data. The first four rows comprise estimates with ‘change in deforestation’ as dependent variable. Including all observations and offense categories (see also Table 2) we obtain a significant ATT of −6.5 ha (baseline estimation). Inspected grid cells exhibit on average 48 hectares of deforestation, i.e. inspections reduce deforestation by 14% on average.

**Table 4:** Average effect of inspections in inspected grid cells.

	ATT	AI SE	AI p-value	Number of treated grid cells
*Dependent: Δ Deforestation*				
**All offense categories all observations**	**−6.514**	**2.433**	**0.0074**	**2786**
**Only deforestation offenses**	**−8.466**	**5.052**	**0.0938**	**595**
**Only deforestation patches >20 ha**	**−8.631**	**4.579**	**0.0595**	**595**
Only deforestation patches <20 ha	0.164	1.859	0.9296	595
*Dependent: Deforestation in t+1*				
All offense categories	−0.166	2.368	0.944	2786
Only deforestation offenses	−1.187	4.822	0.806	595
Only deforestation patches >20 ha	−4.286	4.257	0.314	595
**Only deforestation patches <20 ha**	**3.099**	**1.758**	**0.078**	**595**
*Dependent: share of deforestation patches >20 ha*				
**All offense categories**	**0.013**	**0.008**	**0.089**	**2786**
Only deforestation offenses	0.024	0.018	0.179	595

If we restrict our analysis to inspections that explicitly followed up on illegal deforestation ATT estimates are higher than in the baseline estimation. Here inspections result in a 8.5 ha average reduction of deforestation in the subsequent year, but standard errors are too large to conclude that “pure” deforestation fines are more effective than all fines on average.

In rows 4 and 5, we test whether the effect of treatment differs for large as opposed to small deforestation patches. We differentiate between the scale of deforestation by summing up the total newly deforested area within a grid cell that comes from patches that are smaller vs. larger or equal to 20 ha, respectively. The threshold of 20 ha was chosen based on the fact that IBAMA enforcement agents are guided by the low resolution DETER monitoring system when in the field. DETER is considered accurate in detecting deforestation patches larger than 25 hectares. The high and significant ATT for patches that are larger than 20 ha suggests that inspections deter above all larger scale deforestation. Avoidance behavior, as suggested in section 2, would result in a positive ATT for small deforestation patches (<20 ha), but our estimated effect on change in small-scale deforestation is not significant. This particular result indicates, nonetheless, that the current targeting strategy is at least ineffective with regard to small-scale deforestation.

ATT estimates using absolute deforestation as an alternative dependent variable (rows 5–8 in Table 4) show expected signs, but only the positive effect on small-scale deforestation is significant. Although this latter effect seems to confirm the presence of avoidance behavior, our last two ATT estimates with “share of large scale deforestation” as dependent variable (rows 9–10) are positive and significant in one case. The analyses in Table 4 are thus inconclusive with respect to the effects of field inspections on deforestation patterns, but clearly support earlier research in terms of the overall effectiveness of law enforcement also at the local scale.

### Post-matching analyses

Table 5 reports the results of running weighted least squares regressions with the matched data set, where matching weights are used to account for multiple matches of treatment and control observations. Model (1) results in an average treatment effect equivalent to the result in row 2 of Table 4. Model (2) disaggregates the binary treatment variable into the actual number of precisely measured inspections of ‘deforestation’ and ‘other’ offenses. Model (3) adds all matching covariates and state effects and model (4) adds state-treatment interaction terms. The disaggregated treatment variables are insignificant in models 2–3, but some of the interaction terms in model 4 are significant. It thus seems that inspections have not generally resulted in lower deforestation in subsequent years and that differences in effect size may exist between states.

**Table 5:** Average treatment effects of inspections and inspection intensity.

*Dependent: change in deforestation*	(1)	(2)	(3)	(4)
**Precise inspections yes/no**	−6.514**			
	(3.279)			
N of precise inspections (deforestation)		−3.620	1.117	−8.608
		(2.216)	(2.087)	(5.386)
N of precise inspections (other)		0.112	−0.075	−1.304
		(0.132)	(0.132)	(1.184)
All other matching covariates including state-level fixed effects	No	No	Yes	Yes
**State x treatment interaction**	No	No	No	**Yes** ^**1**^
Adj. R-squared	0.001	0.001	0.273	0.279
N	6526	6526	6526	6526

*Notes*: All models include a constant. Municipal level clustered standard errors are reported in parentheses. *Significance levels*: ‘**’ *0*.*05;*

^*1*^Some state-treatment interaction terms are significant.

Indeed, a potentially important source of treatment effect heterogeneity relates to state-level environmental governance. Recall from section 2, that the main source of deterrence from IBAMA’s field inspections may not be the fine, but the associated set of cross-compliance measures, such as restricting access to credit and commercialization channels. The effectiveness of these measures largely depends on the willingness and capacity of state-level governments to rigorously implement them. Better integration of federal and state-level initiatives in combating deforestation in the Brazilian Amazon has indeed been a key recommendation of the evaluation of the first phase of the PPCDAM [1].

To further explore treatment effect heterogeneity at the state level we estimate Model 3 separately for each of the six states with large amounts of inspections, namely Acre (AC), Amazonas (AM), Mato Grosso (MT), Pará (PA), Rôndonia (RO), and Roraima (RR) (Table 6). Each state-level regression involved prior Mahalanobis distance matching with the full set of covariates (see Fig. 4). To test whether unobserved factors at the state level affect our results, the same regressions were run with a matched data set, where we restricted control cells to come from the same state as treatment cells (see Table A1 in S1 File). Results are similar.

**Table 6:** State–level average treatment effects of inspection intensity after matching.

*Dependent variable*: *change in deforestation*	(AC)	(AM)	(MT)	(PA)	(RO)	(RR)
Average change in deforestation in treated grid cells (ha)	22.34	7.37	−5.04	−26.61	6.37	−5.83
N of precise inspections (deforestation)	3.568	20.154***	−3.965**	−9.868*	−1.579	−9.590*
	(2.633)	(2.118)	(1.977)	(5.304)	(1.394)	(5.547)
N of precise inspections (other)	−4.556	0.638	1.540**	−1.640	−0.105	0.460
	(4.004)	(0.713)	(0.694)	(1.077)	(0.072)	(1.956)
All other matching covariates	Yes	Yes	Yes	Yes	Yes	Yes
Adj. R-squared	0.343	0.253	0.359	0.301	0.208	0.561
N	402	794	1796	1812	898	350

*Notes*: All models include a constant. Municipal level clustered standard errors are reported in parentheses. *Significance levels*:‘***’ *0*.*01* ‘**’ *0*.*05*‘*’ *0*.*1*

Table 6 suggests clear differences in treatment effects across the six states, with the expected negative effect of deforestation-specific inspections significant in Mato Grosso, Pará, and Roraima (Rondônia after within state matching, see Table A1 in S1 File). Running the same regressions only for change in small-scale deforestation (<20 ha) results in a significant positive effect of inspections on change in deforestation only in Roraima state (Table A2 in S1 File). Thus effects are largely driven by reductions in large-scale deforestation (see also Table A3 in S1 File) and indications of avoidance behavior were only detected in one of the six states. Results are robust for the states of Mato Grosso and Pará, for which we ran counterfactual simulations setting the number of deforestation-specific inspections in both observation periods to zero. For Mato Grosso, we find that 449 precisely measured deforestation specific inspections realized during 2009 and 2010 have reduced 1670 hectares of deforestation. In Pará, 296 precisely measured inspections reduced counterfactual deforestation by 2920 hectares over the same period.

Surprisingly, we find a comparatively large and robust positive effect of deforestation-specific fines on total forest loss in the state of Amazonas, i.e. deforestation seems to have increased in the years after inspections.

A potential behavioral explanation is that offenders, who are used to low levels of sporadic enforcement activities in this state with historically low deforestation rates, perceive enforcement risk as particularly low shortly after inspections. The deterrence literature does indeed repeatedly stress the role of subjective threat perception in determining the outcomes of criminal law enforcement [44]. An alternative explanation, however, is that our analysis fails to capture the deterrence effect of enforcement action at the emerging deforestation frontier at the border between the states of Acre and Amazonas. This particular region has been characterized as a major new speculation frontier, among others as a result of the recent completion of the interoceanic highway connecting Brazil to the Andean countries [45]. If similarly dynamic regions are absent from the pool of available control grid cells, or our set of covariates fails to capture key frontier characteristics, our ATT estimates for Amazonas state are likely to be biased.

To explore whether the strong positive effect of inspections in the state of Amazonas is indeed driven by unobserved frontier dynamics, we ran three additional post-matching regressions (Table 7). Matching was done on the previously used set of covariates plus a frontier region dummy that is 1 if a district lies at the border to the state of Acre (7 out of 62 districts in Amazonas state) and zero otherwise.

**Table 7:** Amazonas state average treatment effects after state-level matching.

*Dependent variable: change in deforestation*	(1)	(2)	(3)
Treatment (yes/no)	7.636*	2.036	12.393
	(4.611)	(3.392)	(11.510)
Treatment / frontier dummy interaction		22.781**	
		(10.274)	
All other matching covariates + frontier dummy	Yes	Yes	Yes
Treatment definition	≥1 insp. per cell	≥1 insp. per cell	≥4 insp. per cell
Adj. R-squared	0.154	0.160	0.248
N	794	794	118
*Significance levels*:‘**’ *0*.*05*‘*’ *0*.*1*			

Model (1) shows that the potentially biased positive average treatment effect remains significant after including the additional matching covariate representing frontier characteristics. Model (2), however, indicates that inspections had no significant effect in other than frontier regions. Finally, model (3) suggests that the unexpected positive treatment effect ceases to be significant when we find appropriate control observations for grid cells that were treated with higher intensity (higher number of fines). Models (2) and (3) thus support our notion that the continuous treatment variable used in the state-level analysis in Table 7 may still be endogenously related to deforestation risk in a way that we are unable to control for in Amazonas state.

## Discussion and Conclusion

We have used a spatial matching approach and post-matching regressions to evaluate the effect of geo-referenced field-based forest law enforcement missions on deforestation in the Brazilian Amazon region at the sub-municipal scale. Our inspection data set covers the last two years (2009–2010) of the implementation horizon of the first Brazilian plan to combat deforestation in the region (PPDCAM), when major reductions in annual forest loss had already occurred (see Fig. 1). In this setting, average treatment effect estimates after matching on a comprehensive set of covariates corroborate the findings of previous studies at the municipal level [9, 29]. Deterrence is thus, at least to some extent, a result of being spatially close to field inspections even at the sub-district scale.

The usual caveats of non-experimental impact evaluation techniques, such as the necessary assumption of unconfoundedness [31], also apply to our matching and post-matching regression analysis. Our strategy is to minimize the risk of unobservable bias by drawing on a comparatively large set of biophysical, socioeconomic, and spatial covariates that include the variables known to influence the spatial allocation of field enforcement operations by the Brazilian Environmental Protection Agency. We also assess the sensitivity of our results to the choice of the spatial grid resolution that determines the size of the observational unit in many evaluations of area-based interventions [25, 28, 46].

Our results suggest that field inspections had a particularly strong deterrence effect on large-scale offenses (> 20 ha deforestation patches). Some authors have hypothesized that field-based enforcement could have induced a shift from large to small-scale offenses, which could reflect so called “avoidance behavior” [10]. While we did not find strong evidence supporting the presence of avoidance behavior, our analysis confirms that inspections have been largely ineffective in deterring small-scale deforestation.

In post-matching regressions we found the effect of field-based enforcement to vary substantially across states. Focusing on the six states with the highest inspection intensity, we found significant negative effects of inspections on deforestation that were robust across all analyses for two of the tree largest states in the Brazilian Amazon, Mato Grosso (4.0 ha/inspection) and Pará (9.9 ha/inspection). Based on inspection cost estimates in Börner et al. [23] (3500 USD per inspection on average) and assuming 300 tons of carbon dioxide emissions on average per hectare of deforestation, we estimate that field-based enforcement was cost-effective at break-even carbon prices ranging from 1.2 to 3.0 USD per ton of carbon dioxide in Pará and Mato Grosso, respectively.

In most other states our results suggest that deforestation has at least on average remained unaffected by *in-situ* enforcement action. An unexpected positive effect of field inspections on deforestation in Amazonas state is driven by a small number of frontier districts (7 out of 62) and likely biased.

Further research is needed to disentangle the underlying mechanisms of field-based enforcement effectiveness [47]. It becomes clear, though, that field inspections are merely an incentive delivery instrument and thus not universally effective deterrents. Where governance mechanisms at the state level fail to secure legal follow-up on enforcement campaigns, deterrence is likely to be minimal unless federal agencies significantly raise fine collection rates.

The surge in targeted field-based enforcement operations after the launch of the PPDCAM in 2004 has evidently helped to stabilize deforestation rates at historically low levels. Clearly, regular field-based inspections are still needed to maintain the current level of deterrence. However, a comprehensive post-crackdown strategy to combat illegal deforestation in the region will probably require a more diversified set of measures that align field-based enforcement with effective legal follow-up as well as regionally targeted policies to encourage sustainable land use at the forest margins.

## Supporting Information

S1 File Appendix SI 1. (DOCX)

S1 Dataset Supplementary Data. (ZIP)
