# Supplementary material for: Post-Crackdown Effectiveness of Field-Based Forest Law Enforcement in the Brazilian Amazon
Source: PLoS One. 2015 Apr 15;10(4):e0121544. doi: 10.1371/journal.pone.0121544 (PMC4398318; doi:10.1371/journal.pone.0121544)
Supplement: S1 File — (DOCX) [file pone.0121544.s001.docx]

**APPENDIX: SI 1**

**Table A1: State–level average treatment effects of inspection intensity after matching within states**

| *Dependent variable: change in deforestation* | (AC) | (AM) | (MT) | (PA) | (RO) | (RR) |
| --- | --- | --- | --- | --- | --- | --- |
| Average change in deforestation in treated grid cells (ha) | 22.34 | 7.37 | -5.04 | -26.61 | 6.37 | -5.83 |
| N of precise inspections (deforestation) | 1.934 | 19.003*** | -3.345* | -9.668* | -2.242** | -11.431 |
|  | (2.729) | (2.242) | (1.828) | (5.392) | (0.920) | (7.149) |
| N of precise inspections (other) | -4.892 | -0.103 | 1.722** | -1.960* | -0.089 | -0.485 |
|  | (3.572) | (1.024) | (0.686) | (1.060) | (0.088) | (1.935) |
| All other matching covariates | Yes | Yes | Yes | Yes | Yes | Yes |
| Adj. R-squared | 0.293 | 0.183 | 0.387 | 0.284 | 0.229 | 0.575 |
| N | 402 | 794 | 1800 | 1812 | 898 | 346 |

*Notes:* All models include a constant. Municipal level clustered standard errors are reported in parentheses. *Significance levels: ‘***’ 0.01 ‘**’ 0.05‘*’ 0.1*

**Table A2: State–level average treatment effects of inspection intensity on small-scale deforestation after matching within states**

| *Dependent variable: change in deforestation (< 20 ha)* | (AC) | (AM) | (MT) | (PA) | (RO) | (RR) |
| --- | --- | --- | --- | --- | --- | --- |
| Average change in deforestation in treated grid cells (ha) | 22.34 | 7.37 | -5.04 | -26.61 | 6.37 | -5.83 |
| N of precise inspections (deforestation) | 0.368 | 3.126* | -0.866 | -0.335 | 0.361 | 2.888* |
|  | (2.239) | (1.675) | (0.686) | (2.046) | (0.361) | (1.751) |
| N of precise inspections (other) | 0.051 | 0.311 | 0.420*** | -0.796** | 0.014 | -1.628 |
|  | (1.742) | (0.386) | (0.149) | (0.375) | (0.052) | (1.041) |
| All other matching covariates | Yes | Yes | Yes | Yes | Yes | Yes |
| Adj. R-squared | 0.300 | 0.075 | 0.105 | 0.205 | 0.061 | 0.438 |
| N | 402 | 794 | 1796 | 1812 | 898 | 350 |

*Notes:* All models include a constant. Municipal level clustered standard errors are reported in parentheses. *Significance levels: ‘***’ 0.01 ‘**’ 0.05‘*’ 0.1*

**Table A3: State–level average treatment effects of inspection intensity on large-scale deforestation after matching within states**

| *Dependent variable: change in small-scale deforestation (> 20 ha)* | (AC) | (AM) | (MT) | (PA) | (RO) | (RR) |
| --- | --- | --- | --- | --- | --- | --- |
| Average change in deforestation in treated grid cells (ha) | 22.34 | 7.37 | -5.04 | -26.61 | 6.37 | -5.83 |
| N of precise inspections (deforestation) | 3.200** | 17.028*** | -3.099 | -9.533** | -1.940 | -12.478** |
|  | (1.627) | (1.548) | (1.917) | (4.442) | (1.195) | (4.835) |
| N of precise inspections (other) | -4.607 | 0.327 | 1.120* | -0.844 | -0.119** | 2.088 |
|  | (3.245) | (0.531) | (0.636) | (0.804) | (0.053) | (1.915) |
| All other matching covariates | Yes | Yes | Yes | Yes | Yes | Yes |
| Adj. R-squared | 0.300 | 0.261 | 0.348 | 0.271 | 0.210 | 0.490 |
| N | 402 | 794 | 1796 | 1812 | 898 | 350 |

*Notes:* All models include a constant. Municipal level clustered standard errors are reported in parentheses. *Significance levels: ‘***’ 0.01 ‘**’ 0.05‘*’ 0.1*
